# A Quality Improvement Project to Improve Staff Confidence in Managing Incidences of Patient Violence and Aggression on the Neurosciences Wards

**DOI:** 10.1192/bjo.2024.361

**Published:** 2024-08-01

**Authors:** Isabel Staffurth, Rahima Hoque, Bea Duric, Marianna Rogowska, Sotiris Posporelis

**Affiliations:** 1 King's College London, London, United Kingdom; 2 Institute of Psychiatry, Psychology and Neuroscience, London, United Kingdom

## Abstract

**Aims:**

Incidents involving patient violence and aggression are a common occurrence on the neurosciences wards. Many staff do not know how to de-escalate or manage such incidents, leaving them vulnerable and unsupported. This project was designed to increase mean staff confidence by at least 2 points (on a scale of 1–10) regarding confidence and satisfaction in managing patient violence during a 6-week period.

**Methods:**

Using Plan-Do-Study-Act (PDSA) quality improvement methodology, we carried out a preliminary survey on 2 neurosciences wards. Multidisciplinary staff were interviewed about their confidence (on a scale of 1–10) in managing violence. The survey and interview assessed which measures were already in place on the wards, such as Datix reporting and referral pathways. The first intervention focused on preventing patient violence with informative posters on referral pathways and verbal de-escalation techniques; these were distributed throughout the wards and staff were notified via email. Follow-up surveys were collected, enquiring whether staff had seen the posters and how their confidence levels have changed. The second intervention was implemented 2 weeks later and focused on post-incident support. We distributed leaflets on Critical Incident Staff Support and sent an email link to a verbal de-escalation playlist. Follow-up surveys were collected again to track changes in staff confidence and satisfaction. Weekly electronic clinical record searches were recorded to track the number of incidences of patient aggression during the same 6-week period.

**Results:**

Staff confidence (N = 24) in verbally de-escalating violence and aggression increased by 1.1 and 1.75 points for Wards A and B, respectively. Of the 6 staff members who were followed up, only 1 experienced a 2-point increase from baseline in confidence in verbal de-escalation; 1 staff member experienced a 1-point increase, 3 experienced no change, and 1 reported a 1-point decrease. Staff satisfaction in management of violence on their wards increased by 1.04 and 1.75 points for Wards A and B, respectively. Staff confidence in knowing which team to refer patient violence to increased by 1.167 and 1.07 points for Wards A and B. Incidences of patient violence reported on EPIC decreased by 8 episodes for Ward B and increased by 2 episodes for Ward A.

**Conclusion:**

Low-cost, simple intervention techniques are largely ineffective in improving staff confidence in handling violence. During verbal feedback, most staff agreed that training and simulation-based days would be useful. Ward managers should seek to include well-structured training to improve staff confidence.

